# Transfer learning-assisted 3D deep learning models for knee osteoarthritis detection: Data from the osteoarthritis initiative

**DOI:** 10.3389/fbioe.2023.1164655

**Published:** 2023-04-13

**Authors:** Pauline Shan Qing Yeoh, Khin Wee Lai, Siew Li Goh, Khairunnisa Hasikin, Xiang Wu, Pei Li

**Affiliations:** ^1^ Department of Biomedical Engineering, Universiti Malaya, Kuala Lumpur, Malaysia; ^2^ Faculty of Medicine, Universiti Malaya, Kuala Lumpur, Malaysia; ^3^ School of Medical Information and Engineering, Xuzhou Medical University, Xuzhou, China; ^4^ Information Department, Affiliated Hospital of Xuzhou Medical University, Xuzhou, China

**Keywords:** convolutional neural network, deep learning, disease classification, knee osteoarthritis, magnetic resonance imaging

## Abstract

Knee osteoarthritis is one of the most common musculoskeletal diseases and is usually diagnosed with medical imaging techniques. Conventionally, case identification using plain radiography is practiced. However, we acknowledge that knee osteoarthritis is a 3D complexity; hence, magnetic resonance imaging will be the ideal modality to reveal the hidden osteoarthritis features from a three-dimensional view. In this work, the feasibility of well-known convolutional neural network (CNN) structures (ResNet, DenseNet, VGG, and AlexNet) to distinguish knees with and without osteoarthritis (OA) is investigated. Using 3D convolutional layers, we demonstrated the potential of 3D convolutional neural networks of 13 different architectures in knee osteoarthritis diagnosis. We used transfer learning by transforming 2D pre-trained weights into 3D as initial weights for the training of the 3D models. The performance of the models was compared and evaluated based on the performance metrics [balanced accuracy, precision, F1 score, and area under receiver operating characteristic (AUC) curve]. This study suggested that transfer learning indeed enhanced the performance of the models, especially for ResNet and DenseNet models. Transfer learning-based models presented promising results, with ResNet34 achieving the best overall accuracy of 0.875 and an F1 score of 0.871. The results also showed that shallow networks yielded better performance than deeper neural networks, demonstrated by ResNet18, DenseNet121, and VGG11 with AUC values of 0.945, 0.914, and 0.928, respectively. This encourages the application of clinical diagnostic aid for knee osteoarthritis using 3DCNN even in limited hardware conditions.

## 1 Introduction

Knee osteoarthritis (OA) is the most prevalent type of arthritis and has negatively impacted approximately 5% of the world population (Morales Martinez et al., 2020). This disease is highly prevalent among the older population (Hossain et al., 2014). It is a progressive disease that leads to impairment and disability in patients, affecting one’s quality of life. This disease is typically diagnosed manually using medical imaging such as X-ray and magnetic resonance imaging (MRI) (Yong et al., 2021a; Teoh et al., 2022).

With the increasing number of publicly available medical datasets, the development of artificial intelligence-based diagnostic models has become more feasible and has emerged rapidly in the recent years. Deep learning algorithms, particularly convolutional neural networks (CNNs), have proven to be effective for analyzing medical images and providing more accurate results with less pre- and post-processing than traditional methods. CNN has been widely applied in a variety of tasks in the medical field, including the identification and segmentation of regions of interest, such as organs and tumors, and the classification and prediction of different diseases. Basically, a CNN takes images as input, and the convolutional layers within are trained to learn the features by assigning weights and biases, allowing the CNN model to improve over time. Due to computational constraints, most deep learning studies from the previous years adopted 2DCNN in most of the computer vision applications of 2D inputs (Klaiber et al., 2021). The 2DCNN uses a 2D convolutional kernel that only collects spatial information in two dimensions. Hence, conventionally, to accommodate 3D data such as MRI in 2DCNN, the appropriate slice and orientation have to be selected as input (Chang et al., 2020). However, with this approach, the information along the third dimension is neglected. The 3DCNN overcomes the problematic slice selection process, allowing 3D computer vision tasks to be less tedious by accepting the whole 3D MRI volume (Khagi and Kwon, 2020). In comparison to the 2DCNN, 3DCNN captures the spatial information in all three dimensions, including spatial connections between 2D slices, allowing a more comprehensive view of the volumetric data to extract more distinguishable representations. However, obtaining high-quality annotated medical images is challenging, making it difficult to develop a robust deep learning model. Implementing 3DCNN is harder since 3D training datasets are limited and more computationally expensive (Klaiber et al., 2021).

Transfer learning is a powerful technique that leverages previous knowledge to enhance model performance (Wu et al., 2020). Transfer learning has been a prominent approach to address the medical data scarcity problem using pre-trained models trained on huge datasets to extract useful features for small target datasets. This technique not only uses the weights and biases from pre-trained models but also contributes by saving time and computation resources compared to training a new model from scratch (Kim et al., 2022). The configuration of the transfer learning approach varies according to the models and tasks involved, with the aim to maximize the performance of the target task (Wu et al., 2020; Kim et al., 2022). In the context of 3DCNN tasks, several studies have transformed 2D weights from pre-trained models on the 3DCNN and presented favorable results in their respective tasks (Ebrahimi et al., 2020; Merino et al., 2021).

The majority of the previous CNN approaches in knee osteoarthritis diagnosis are based on 2D plain radiography (Yeoh et al., 2021). Although knee radiographs have a significant role in diagnosing OA, it is well known that it is insensitive in early OA detection, as shown in several recent studies (Tiulpin et al., 2018; Kim et al., 2020a; Nguyen et al., 2020). Nguyen et al. (2020) proposed a novel Semixup algorithm, which achieved a relatively low accuracy for early OA stages. With 500 labeled samples per Kellgren–Lawrence (KL) grade, Semixup achieved 58% accuracy for KL grade 2, whereas with 1,000 labeled samples per KL grade, the model obtained 38% accuracy for KL grade 1. Kim et al. (2020a) used the SE-ResNet algorithm and reported having difficulty in diagnosing KL grade 2. Plain radiography might have low accuracy in detecting early OA because the changes of articular cartilages that are crucial in OA progression assessment are not visible in plain radiography. Alternatively, these changes can be directly visualized by MRI.

While X-ray imaging can visualize bony changes associated with knee OA, MRI provides precise depiction of the knee joint structures, with better contrast for visualizing both bony and soft tissue changes (Faisal et al., 2018; Chang et al., 2020; Yong et al., 2021b; Teoh et al., 2022). The soft tissue change is pertinent for knee OA diagnosis because the shape of knee joint cartilages changes significantly as the disease progresses (Hossain et al., 2015; Hayashi et al., 2019). Since the 2DCNN trained on plain radiography examines one 2D image at a time, it is unable to capture the complex 3D structure of the knee joint as compared to the 3DCNN approach that uses the whole sequence of 3D MRI as a single unit (Guida et al., 2021). Previous studies suggested that 3D MRI may contain intrinsic information that can recognize subtle changes in the knee joint, which contributes to better sensitivity for OA detection as compared to 2D plain radiography (Tolpadi et al., 2020).

Moreover, since knee OA is a 3D complexity that involves the whole joint, MRI will be the ideal modality for OA assessment, having the potential to reveal hidden OA structures using the 3DCNN. This allows better interpretation of the condition of the knee through volumetric analysis. This is also supported by a recent study by Guida et al. (2021), who showed that 3DCNN combined with MRI volumes can outperform 2DCNN on plain radiography in knee OA diagnosis. Despite these advances, there is a gap of knowledge in the feasibility of 3DCNN in knee OA detection, which is still subject to intensive research.

The purpose of this study is to fill the void by leveraging transfer learning of 2D pre-trained weights in the 3DCNN in medical imaging, particularly for knee OA diagnosis. In this work, we focus on binary classification to identify knees with OA. With interest in finding the best model for 3D knee osteoarthritis detection, the effectiveness of transfer learning is validated among different CNN architectures. This study takes the advantage of pre-trained models in distinguishing knees with and without OA by using their weights. This study enhances the existing research by evaluating and comparing 13 models trained from scratch and ImageNet (Deng et al., 2009) pre-trained weights, respectively, with different performance metrics.

Motivated by the performance of deep learning models and the potential of transfer learning in medical images, 13 end-to-end automated knee OA diagnosis systems are developed. To the best of our knowledge, this is the first work that includes the exploration of the potential and comparative analysis of 3D deep learning models and transfer learning in knee osteoarthritis detection. The main contributions of this work are as follows:

1. We conducted a comparative study of 13 different feature extractors from commonly used CNN models on knee OA detection.
2. Our work investigates the ability of the models to learn relevant features from whole MRI scans through an end-to-end deep learning approach.
3. Contrary to other comparative studies, the potential of transfer learning on the classification performance of all the involved models was investigated. The result showed that fine-tuned models can achieve higher accuracy than training from scratch, which is effective when the available training dataset is limited.
4. We investigated the robustness of different CNN architectures from two different training approaches (scratch vs. transfer learning) by assessing the evaluation metrics and comparing them based on the models’ size and number of parameters. The result demonstrated that shallower networks can be more efficient by producing desirable outcomes with reduced computational cost (memory usage and number of parameters).

## 2 Materials and methods

### 2.1 Dataset

Four hundred knee MRI volumes of 3D sagittal double-echo steady-state (DESS) scans from Siemens 3T Trio systems were used in this study. All the MRI volumes were obtained from the publicly available Osteoarthritis Initiative (OAI) dataset (https://nda.nih.gov/oai/). OAI is a multicenter longitudinal study of 4,796 participants with comprehensive clinical data, imaging data, and analysis from different time points. All volumes used in this study are collected from 400 different subjects from the baseline timepoint. All MRI volumes were converted to the Neuroimaging Informatics Technology Initiative (NIfTI) standard. The volumes were transformed to a right-anterior-superior coordinate frame, resized to 160 × 160×160, Z-normalized, and standardized, which rescales the intensity values of the voxels from 0 to 1 with a uniform distribution. All datasets were randomly classified into training, validation, and test datasets with a 7:2:1 split.

The Kellgren–Lawrence (KL) grade is one of the most common OA severity grading systems to diagnose patients with knee OA in the clinical setting (Guida et al., 2021). To perform a 2-class classification, the KL grades of each volume were retrieved from OAI and categorized into two classes based on the occurrence of OA. As recommended in a previous study by Norman et al. (2019), KL grades of 0 and 1 were grouped into “No OA” or class 0, whereas KL grades of 2, 3, and 4 were grouped into “OA” or class 1. Table 1 shows the distribution of the dataset used in this study.

**TABLE 1:** Distribution of dataset.

Dataset	No of volume samples in each class	
	No OA	OA
Training	87	193
Validation	32	48
Test	16	24
Total MR volumes used in this study	135	265
	400	

### 2.2 Study design

**FIGURE 1:**
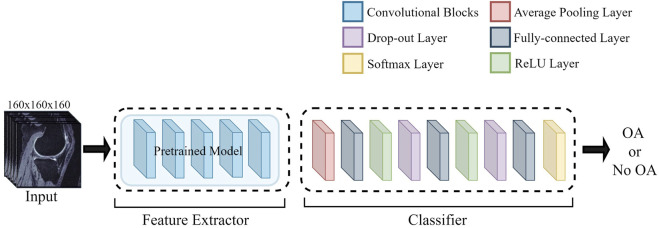
General neural network architecture used in this study.

The overall study provides a comparative overview of different deep learning architectures training from scratch and training from pre-trained weights. This study makes use of a transfer learning approach for knee OA detection by using the pre-trained weights as initial weights and further fine-tuning the model. The components used to construct the complete neural network model are discussed in the following sections. Figure 1 shows the general architecture.

Generally, a neural network is composed of two different sets of layers. The first set of the model layer that carries out feature extraction from the input volume is known as the feature extractor. The other set of layer that comprises fully connected layers to extract features and perform class prediction is known as the classifier. As shown in Figure 1, the feature extractor of the pre-trained models is adapted in this study along with their pre-trained weights only, without the classifier of the pre-trained models. The classifier used by all architectures in this study is identical; it consists of an average pooling layer before three fully connected layers (layer output: 128, 32, 2), with the last final output of two classes and a SoftMax layer. The first two fully connected layers are followed by a ReLU layer and a dropout layer of 0.5.

### 2.3 Pre-trained model architecture and transfer learning

Conceptually, the feature extractor consists of convolutional layers and pooling layers to detect and extract features of the image to be forwarded to the classifier. In this study, 13 different convolutional neural networks from four different architectures [ResNet (He et al., 2016), DenseNet (Huang et al., 2017), VGG (Simonyan and Zisserman, 2014), and AlexNet (Krizhevsky, 2014)] were investigated. All the VGG networks implemented in this study are models with batch normalization layers, which help to deal with the vanishing gradient.

All the models were previously proposed in 2D (Krizhevsky, 2014; Simonyan and Zisserman, 2014; He et al., 2016; Huang et al., 2017), and the 3D models used in this research were constructed in 3D by replacing the 2D operations of the original model with their 3D counterparts. The implemented models were modified to accept the 3D DESS MRI as input.

The models with two types of initial weights, 1) ImageNet (Deng et al., 2009) pre-trained initial weights (transfer learning) and 2) randomly assigned initial weights (training from scratch), were trained and compared. There are different approaches for transfer learning as it is an adaptable application of transferring the previously learned parameters (weights and biases) from pre-trained models to entirely new models that solve a related or novel problem. The pre-trained weights used in this study are pre-trained weights trained with ImageNet (Deng et al., 2009) provided in the torchvision package from the PyTorch (Paszke et al., 2019) library. A weight initialization scheme is implemented, where the pre-trained weights are used as the initial weights of the training process of the 3D models. This is also known as fine-tuning (Tajbakhsh et al., 2016).

**FIGURE 2:**
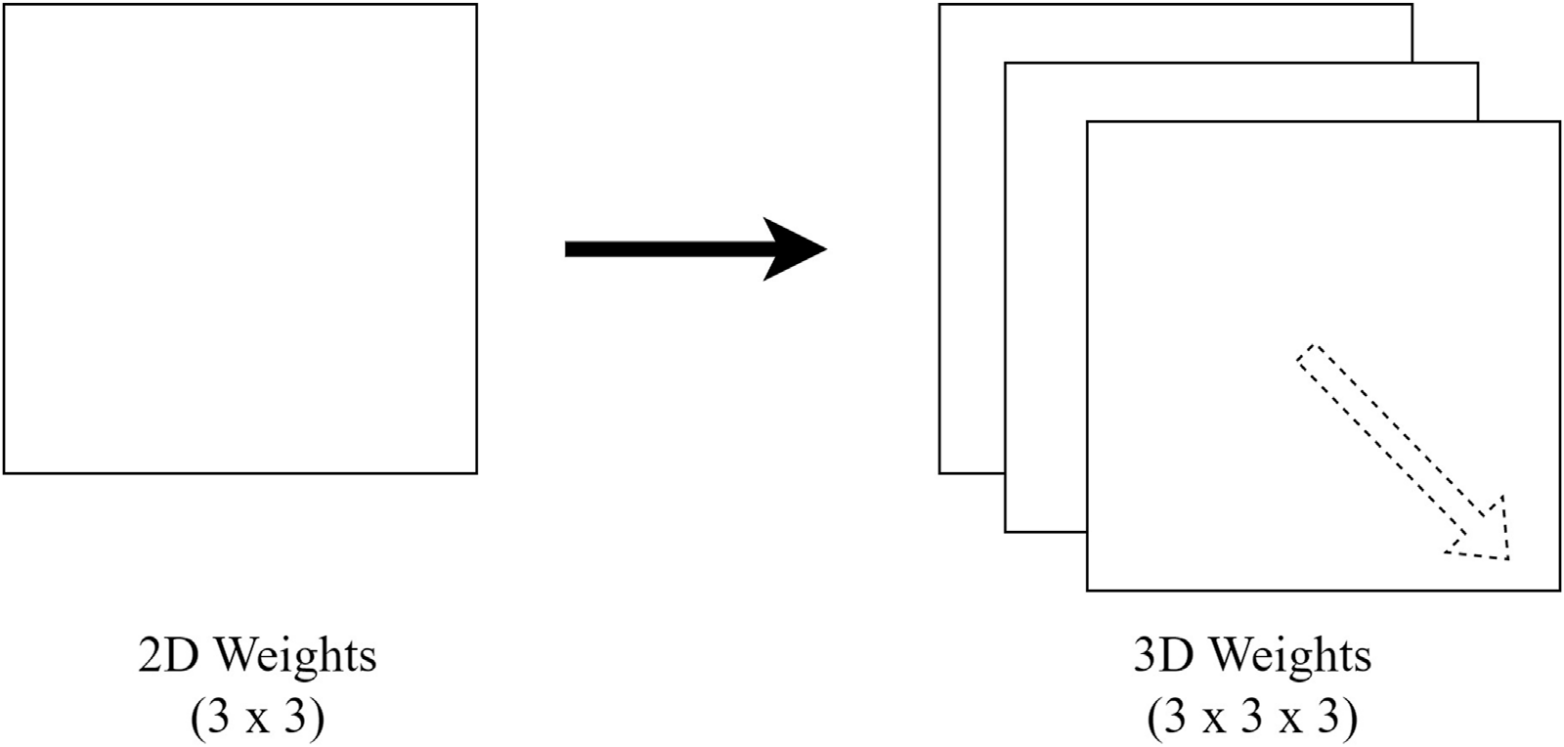
Replication of 2D weights along the third dimension.

Since the models implemented in this study are in 3D, but the available pre-trained weights are in 2D, to transfer the learnable parameters, extrusion (Merino et al., 2021) of weights is performed, where the 2D weights are duplicated along the third axis to transform them into 3D. Figure 2 visualizes the repeated 2D weights along the third dimension.

### 2.4 Training specifications

The same training strategy and hyperparameters were applied for all model training sessions in this study, regardless of the training method (transfer learning or training from scratch). The input sizes used for all training sessions are kept identical, as specified earlier in the dataset section. To train the neural networks, the models are optimized with an ADAM optimizer with a learning rate of 1e-5. The learning rate is decided after some preliminary testing, where a higher learning rate may negatively affect the performance of the models. The optimal learning rate chosen allows the model to learn effectively to make progress toward the optimal weights and biases. The models were trained with a batch size of four and a drop out of 0.5, with a maximum of 50 epochs. We selected 50 as the suitable amount for maximum epoch as most of the models showed convergence below 50 epochs. Since the training dataset has a class imbalance problem, weighted random sampler (implemented by PyTorch (Paszke et al., 2019)), an oversampling technique based on class weights, was used to allow equal class distribution during model training. Early stopping was applied, where the training stops if the validation loss does not reduce over the preceding 10 epochs to prevent overfitting of the models. The loss function used is the cross-entropy loss function. All training sessions were implemented using PyTorch on central processing unit Intel Xeon W-2225 CPU and graphics processing unit (GPU) NVIDIA RTX A6000 with random access memory (RAM) of 32.0 GB.

### 2.5 Evaluation metric

The classification performance of the models was evaluated on the test dataset and analyzed in terms of evaluation metrics. The evaluation metrics were based on the confusion matrix characteristics, which are true positives (TPs), true negatives (TNs), false positives (FPs), and false negatives (FNs). In this work, the positive class is defined as the class of interest, where TP refers to the number of samples belong to that specific class whereas FP refers to the number of samples that do not belong to that specific class but are labelled as such by the model. The evaluations used in this study are as follows:

#### 2.5.1 Accuracy (balanced accuracy)

Acknowledging that our dataset used is imbalanced, balanced accuracy is used instead of the commonly used accuracy metric. Balanced accuracy is also defined as the average of recall obtained in each class and is the ratio of correctly classified samples to the total number of samples in that particular class. In binary classification, it is defined as follows (Guyon et al., 2015):

$$Balanced\;Accuracy=\frac{1}{2}\left(\frac{TP}{TP+FN}+\frac{TN}{TN+FP}\right).$$

#### 2.5.2 Precision

It is the measure of correctly predicted samples out of all predicted positive samples.

$$Precision=\frac{TP}{TP+FP}.$$

#### 2.5.3 F1 score

It is a harmonic average between precision and recall with a minimum value of 0 and a maximum value of 1.

$$F1=\frac{2TP}{2TP+FP+FN}.$$

#### 2.5.4 Area under receiver operating characteristics curve

AUC is the area under the plot of the true positive rate (TPR) against the false positive rate (FPR). It demonstrates the ability of a model to distinguish two classes.

$$TPR=\frac{TP}{TP+FN}.$$

$$FPR=\frac{FP}{FP+TN}.$$

## 3 Results

A range of pre-trained models, which consist of different architectural configurations of ResNet, DenseNet, VGG, and AlexNet, with and without pre-trained weights, were compared in this study. The models are classified based on their architectural innovations: 1) ResNet with residual modules involving shortcut connections; 2) DenseNet, which is composed of dense blocks; and 3) VGG, which is made of repeating structures of convolution layers. Since AlexNet is also made up of stacked convolutional layers without residual or dense blocks, it is categorized under VGG in the following sections for comparison. The overview of the performance of all models is summarized in Table 2, Table 3, and Table 4. The accuracy per class is provided and is defined as the ratio of correctly classified samples per class. For precision, F1 score and AUC metrics, the macro-average of the performance metrics are presented as the overall performance of the model. Both classes (No OA and OA) in this study are treated equally while evaluating the overall performance. The best performance metrics among the architectures are in bold.

**TABLE 2:** Performance comparison of ResNet variants.

Transfer learning-based model (pre-trained weights)								
	Parameters (M)	Memory (MB)	Accuracy		Balanced accuracy	Precision	F1 score	AUC
			No OA	OA				
ResNet18	33.230	132.919	0.625	1.000	0.812	**0.900**	0.829	**0.945**
ResNet34	63.539	254.158	0.875	0.875	**0.875**	0.868	**0.871**	0.925
ResNet50	46.422	185.686	0.563	0.958	0.760	0.833	0.772	0.900
ResNet101	85.468	341.871	0.875	0.500	0.688	0.698	0.649	0.736
ResNet152	117.626	470.506	0.625	0.917	0.771	0.810	0.780	0.901

Scratch-based model (random weights)								
	Parameters (M)	Memory (MB)	Accuracy		Balanced accuracy	Precision	F1 Score	AUC
			No OA	OA				
ResNet18	33.230	132.919	0.250	0.958	0.604	**0.729**	0.580	**0.763**
ResNet34	63.539	254.158	0.625	0.792	**0.708**	0.713	**0.710**	0.763
ResNet50	46.422	185.686	0.500	0.625	0.563	0.561	0.562	0.529
ResNet101	85.468	341.871	0.125	0.875	0.500	0.500	0.451	0.421
ResNet152	117.626	470.506	0.000	1.000	0.500	0.300	0.375	0.518

The highest performance metrics values among the architectures are in bold.

**TABLE 3:** Performance comparison of DenseNet variants.

Transfer learning-based model (pre-trained weights)								
	Parameters (M)	Memory (MB)	Accuracy		Balanced accuracy	Precision	F1 score	AUC
			No OA	OA				
DenseNet121	11.378	45.512	0.563	0.958	0.760	**0.833**	0.772	**0.914**
DenseNet169	18.760	75.040	0.625	0.917	**0.771**	0.810	**0.780**	0.913
DenseNet201	25.581	102.324	0.625	0.875	0.750	0.774	0.757	0.891

Scratch-based model (random weights)								
	Parameters (M)	Memory (MB)	Accuracy		Balanced accuracy	Precision	F1 Score	AUC
			No OA	OA				
DenseNet121	11.378	45.512	0.5	0.833	**0.667**	**0.691**	**0.670**	**0.798**
DenseNet169	18.760	75.040	0.125	0.917	0.521	0.556	0.467	0.542
DenseNet201	25.581	102.324	0.000	0.958	0.479	0.295	0.365	0.530

The highest performance metrics values among the architectures are in bold.

**TABLE 4:** Performance comparison of AlexNet and VGG variants.

Transfer learning-based model (pre-trained weights)								
	Parameters (M)	Memory (MB)	Accuracy		Balanced accuracy	Precision	F1 score	AUC
			No OA	OA				
AlexNet	15.119	60.476	0.000	1.000	0.500	0.300	0.375	0.500
VGG11	50.141	200.565	0.750	0.792	0.771	0.766	0.768	**0.928**
VGG13	50.695	202.779	0.688	0.833	0.760	0.767	0.763	0.914
VGG16	66.624	266.495	0.688	0.875	0.781	0.797	0.787	0.883
VGG19	82.553	330.211	0.688	1.000	**0.844**	**0.914**	**0.860**	0.909

Scratch-based model (random weights)								
	Parameters (M)	Memory (MB)	Accuracy		Balanced accuracy	Precision	F1 Score	AUC
			No OA	OA				
AlexNet	15.119	60.476	0.000	1.000	0.500	0.300	0.375	0.504
VGG11	50.141	200.565	0.688	0.750	0.719	0.715	0.716	**0.870**
VGG13	50.695	202.779	0.563	0.833	0.698	0.717	0.703	0.811
VGG16	66.624	266.495	0.750	0.958	**0.854**	**0.888**	**0.865**	0.869
VGG19	82.553	330.211	0.625	0.958	0.792	0.851	0.804	0.866

The highest performance metrics values among the architectures are in bold.

### 3.1 Comparison analysis among different architectures

The comparative analysis of the ResNet variants is presented in Table 2. Among the ResNet variants, ResNet18 and ResNet34 showed better performance, with and without transfer learning. With transfer learning, ResNet18 achieved an overall AUC score of 0.945, which is the highest among all architectures. The ability of ResNet18 to distinguish between OA and No OA is the best, with an accuracy score of 1.0 in OA detection. ResNet34 obtained the highest overall balanced accuracy (0.875) and F1 score (0.871). It is worth noting that ResNet18 and ResNet34 trained from scratch obtained a relatively good performance compared to their other variants with transfer learning, showing the models’ potential in knee OA detection even without pre-trained weights.

Table 3 shows the comparative analysis of the DenseNet variants. Transfer learning-based DenseNet121 obtained the highest overall AUC (0.914) and precision (0.833) scores, whereas transfer learning-based DenseNet169 achieved the highest overall balanced accuracy (0.771) and F1 score (0.780) among the DenseNet variants.

For all the architectures of the ResNet and DenseNet variants, the models initialized with pre-trained weights performed better than those with random weights. However, from the comparative analysis presented in Table 4, VGG16 trained from scratch performed better than the variant with pre-trained weights. Scratch-based VGG16 achieved a good score in balanced accuracy (0.854), precision (0.888), and F1 score (0.865) metrics. With transfer learning, VGG19 outperformed the other variants with a balanced accuracy of 0.844, F1 score of 0.860, and precision of 0.914, with AUC slightly lower than that of VGG11 (0.909 vs. 0.928.) The performance of AlexNet in this study is the worst among all architectures, with or without transfer learning.

### 3.2 Comparison with another existing study

A comparative evaluation of the best-performing models in this study against a current existing study is presented in Table 5. The performance metrics achieved by the models in our study that exceeded those in the existing study (Guida et al., 2021) are in bold. Since studies on 3D MRI in classification task for OA diagnosis is really limited as it is an emerging research field, only one similar study on knee OA classification is adopted for comparison in this study.

**TABLE 5:** Comparison between the best-performing models in this study and the model in an existing study.

	Balanced accuracy	F1 score	AUC
ResNet18 (TL)	0.812	0.829	**0.945**
ResNet34 (TL)	**0.875**	**0.871**	**0.925**
DenseNet121 (TL)	0.760	0.772	**0.914**
VGG11 (TL)	0.771	0.768	**0.928**
VGG19 (TL)	**0.844**	**0.860**	0.909
VGG16 (scratch)	**0.854**	**0.865**	0.869
Guida et al. (2021)	0.817	0.831	0.911

The performance metrics values that outperformed those in the existing study (Guida et al. (2021)) are in bold.

**FIGURE 3:**
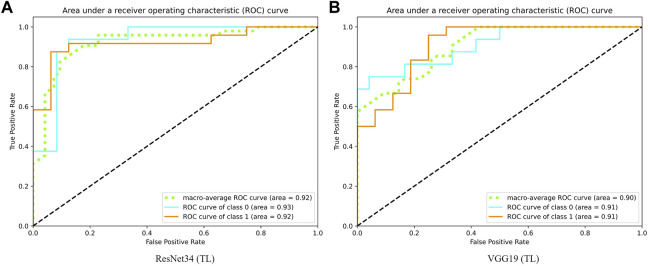
Graph of area under receiver operating characteristic curve of **(A)** ResNet34 and **(B)** VGG19.

Guida et al. (2021), the authors of the existing study, also used the OAI datasets, where the quality and characteristics of the dataset, such as original pulse sequence, resolution, and acquisition plane, are similar to those in our study. This allows a relatively fair comparison among the studies. Guida et al. (2021) reported an overall accuracy of 0.817, F1 score of 0.831, and AUC of 0.911 using their proposed model, with a total of 1,100 data included in their study. The successful knowledge transfer from ImageNet (Deng et al., 2009) to medical imaging applications, specifically OA, is exhibited excellently, especially in ResNet34 and VGG19. The AUC curves for the best-performing transfer learning-based models, 1) ResNet34 and 2) VGG19, which are presented in Figure 3. With transfer learning, ResNet34 in this study has surpassed the results of Guida et al. (2021) in terms of all three evaluation metrics. A comparable performance has been achieved by other transfer learning models, where they exceeded the existing study (Guida et al., 2021) in terms of AUC, whereas VGG16 trained from scratch outperformed the study in terms of accuracy (0.854 vs. 0.817) and F1 score (0.865 vs. 0.831).

## 4 Discussion

In this study, different architectures of the convolutional neural network are analyzed and compared based on their ability to detect knees with or without knee osteoarthritis from 3D MRI volumes. The feasibility of pre-trained weights is well-demonstrated, where generally, the accuracy, F1 score, and AUC score obtained for transfer learning-based models are better than those of models trained from scratch. This result supports a previous finding on different applications (Ebrahimi et al., 2020), validating the expediency of transferring 2D weights to 3D models. Training from scratch is usually not optimized and is computationally expensive, but transfer learning has made the application of the models for knee OA detection possible, even with a relatively smaller number of samples involved as compared to existing studies. It is anticipated and proven that transfer learning will be beneficial for other medical imaging applications (Kim et al., 2020b).

One of the interesting findings in this study is that it differs from results from other medical imaging studies, where the AlexNet performs well in their classification study, such as on chest radiographs (Bressem et al., 2020) and brain MRI (Maqsood et al., 2019) scans. This, once again, shows that the development of a CNN is problem-dependent and not a one-model-fits-all solution. Moreover, results for AlexNet are far below expectations in knee OA detection, even with the use of transfer learning.

Our study has presented the classification of knee osteoarthritis with minimal pre-processing computations and efforts. Unlike previous studies that used segmented images or volumes for classification tasks (Nunes et al., 2019; Pedoia et al., 2019), this study has presented the ability of 3DCNN models to extract sufficient insights from entire unsegmented volumes to classify knee OA. Furthermore, to allow the models to encode the spatial patterns of 3D knee MRI completely, the entire MRI was involved for training without cropping or segmenting the volume. Although we perform resizing of the volume due to GPU memory restrictions, the input volume for the models still comprises all the knee structures included in the original MRI volumes. This approach differs from another recent study of 3DCNN application in knee osteoarthritis, where the authors only used a subregion of the image that comprises the cartilage and joint area (Guida et al., 2021). This makes the system in our study more automatic and robust. Despite the fact that diagnosis usually may be based on a few features of a radiographic image, where the cartilage and the tibiofemoral joint region are certainly indicators of OA, with the collective information of all the knee components in a single knee MR volume, other OA biomarkers such as bone marrow lesions in both tibiofemoral and patellofemoral joints can add value to the training of the osteoarthritis detection model.

**FIGURE 4:**
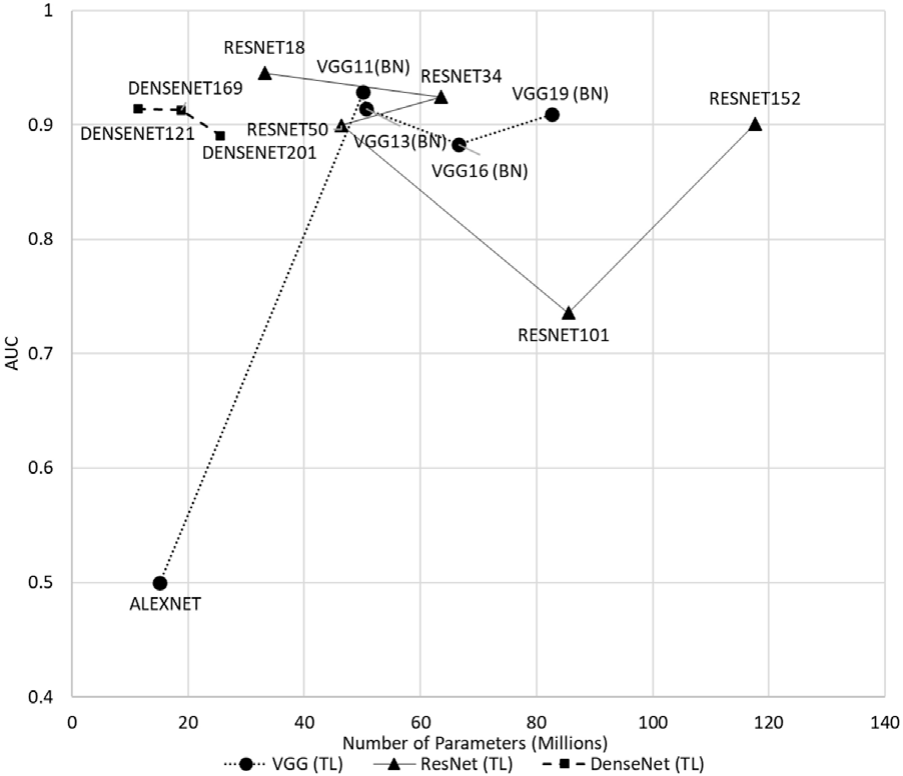
Comparison of all transfer learning-based models in terms of AUC.

Our findings align with a recent study by Bressem et al. (2020), where the usage of deeper architectures did not result in a significant performance increase for knee osteoarthritis detection. As illustrated in Figure 4, shallow networks such as ResNet18, DenseNet121, and VGG11 achieved an AUC of scores 0.945, 0.914, and 0.928, respectively, exhibiting an excellent ability for osteoarthritis detection, as compared to their deeper counterparts. The tendency of showing lower AUC in the deeper counterparts is shown among all architectures, with and without transfer learning. Current CNN development is getting deeper, where with the increasing complexity of the models, the computational resources needed will be heavier. This finding addresses the complexity of the current 3DCNN, where shallow networks with lower hardware requirements have the potential and should be used fully for different applications, increasing the possibility of more efficient 3DCNN being deployed in real-world scenarios, especially with limited hardware. The deployment of the models in embedded healthcare devices as a clinical decision aid can be an efficient alternative to assist clinicians in knee osteoarthritis diagnosis.

Although the findings obtained are encouraging, there are a few drawbacks in this present work. The models in our studies were not optimized, where all the models are trained under the same condition and hyperparameters, as described in previous sections. The objective of the present work is to provide an overview of a comparison between neural networks with and without transfer learning and not on maximizing the performance of the models. Hyperparameter tuning will indeed improve the model’s performance in terms of accuracy and convergence speed. However, performing hyperparameter optimization is challenging and not feasible in this study as there are a large number of CNNs involved in this study. Determining the ideal hyperparameter in this study will be difficult and time-consuming; however, we intend to optimize the best-performing models in future works. However, it is suggested that this problem might not have a significant impact on the overall findings (Tajbakhsh et al., 2016). Moreover, the number of samples used in this study is relatively less than that in other knee OA research studies due to the available computation resources. Without a doubt, the performance of the models can be enhanced with more training samples. Another limitation of the current work is the lack of generalizability of the developed models to accurately interpret and analyze MRI data from various datasets and sequences. The use of different MRI sequences in research studies has led to a research gap in a generalized approach, where the learned features can be transferable across different sequences, datasets, or populations. Separate testing on completely independent datasets is necessary to validate the generalizability of a deep learning model (Pedoia et al., 2019). The currently available dataset with ground truth is not diverse enough, and hence, it is suggested to gather more ground truth data for a heterogeneous dataset. Conducting larger studies that involve multiple sequences from multiple datasets can confirm our preliminary findings from this study. One of the strategies to develop a generalized approach that is robust to variation among MRI datasets is to use transfer learning to transfer learned features across different MRI datasets. Hence, in order to fully validate the models’ potential in knee osteoarthritis detection, more data from public and private datasets can be incorporated into future works. In addition, MRI’s excellent soft tissue contrast might contribute to the early detection of OA, which is an important stage pertinent to OA diagnosis. This work can be further extended to explore the classification of different OA gradings and identification of early OA.

## 5 Conclusion

The findings concluded that transfer learning-based models are generally more robust and accurate than models trained from scratch. The application of transfer learning in medical imaging has demonstrated its importance by achieving high accuracy in knee osteoarthritis diagnosis, using pre-trained weights from ImageNet as initial weights. Shallower 3D convolutional neural networks such as ResNet18, DenseNet121, and VGG11 achieved comparable results to the deeper networks. This encourages the application of 3D shallow neural networks in medical imaging tasks to allow more efficient training and deployment, especially with limited computation resources.

## Data Availability

The original contributions presented in the study are included in the article/Supplementary Material; further inquiries can be directed to the corresponding authors.
